# Observation of Peripapillary Choroidal Vascularity in Natural Disease Course and After Gene Therapy for Leber's Hereditary Optic Neuropathy

**DOI:** 10.3389/fmed.2021.770069

**Published:** 2021-12-07

**Authors:** Jingwen Jiang, Gongpeng Sun, Qingmei Miao, Bin Li, Dan Wang, Jiajia Yuan, Changzheng Chen

**Affiliations:** ^1^Eye Center, Renmin Hospital of Wuhan University, Wuhan, China; ^2^Department of Ophthalmology, Tongji Hospital, Tongji Medical College, Huazhong University of Science and Technology, Wuhan, China; ^3^Neurophth Biotechnology Co., Ltd. Wuhan, China

**Keywords:** Leber's hereditary optic neuropathy, choroidal thickness, choroidal vascularity, blood flow, gene therapy

## Abstract

**Purpose:** To compare peripapillary choroidal vascularity among Leber's Hereditary Optic Neuropathy (LHON) patients at different stages of natural course and healthy controls using optical coherence tomography (OCT), and to evaluate peripapillary choroidal vascularity changes in LHON patients before and after gene therapy.

**Methods:** 57 LHON patients and 15 healthy controls were enrolled in this prospective clinical study. LHON patients were divided into three duration groups based on stage of disease progression. Both patients and healthy controls underwent OCT scans focused on the optic disc at baseline with Heidelberg Spectralis, and patients underwent OCT at 1, 3, and 6 months after gene therapy. OCT images were converted and binarized using ImageJ software. Choroidal thickness (CT), total choroidal area (TCA), and choroidal vascularity index (CVI) in each quadrant of OCT images were measured to evaluate peripapillary choroidal vascularity.

**Results:** At baseline, the average CT was not significantly different between LHON patients at different stages and between healthy controls (*P* = 0.468). Although average TCA and average CVI were slightly higher in LHON patients at different stages than in healthy controls, the difference was not statistically significant (*P* = 0.282 and 0.812, respectively). After gene therapy, The average TCA at 1 month after gene therapy was significantly higher than that before gene therapy (*P* = 0.003), while no significant differences were found in the average CT or average CVI in LHON patients before and 1,3 and 6 months after gene therapy using pairwise comparisons (all *P* > 0.05).

**Conclusions:** No significant difference was found in choroidal vascularity of LHON patients at different stages and healthy controls. Choroidal vascularity seems to stay stable after gene therapy.

## Introduction

Leber's hereditary optic neuropathy (LHON) is a maternally-inherited mitochondrial disease that leads to acute bilateral loss of visual acuity (VA) and visual field in young adulthood (1). LHON patients with the most common mutation site 11778G >A show the worst VA prognosis, and, nowadays, gene therapy targeting this mutation has proven to be the most promising treatment in our previous studies (2, 3).

Along with mutations in mitochondrial DNA (mtDNA), the dysfunction of the mitochondrial respiratory chain complex 1 leads to impairment of ATP production and increased production of reactive oxygen species (ROS), resulting in loss of retinal ganglion cells (RGC) and, eventually, optic nerve atrophy (4). The advent of optical coherence tomography (OCT) has enabled the visualization of structural features in the eyes of LHON patients, such as the progression of retinal nerve fiber layer (RNFL) and ganglion cell complex (GCC) pathology at different stages of the disease (5–8). Furthermore, retinal and peripapillary retinal capillaries were found to be abnormal in LHON patients using OCT angiography (OCTA) (9, 10).

There have been few investigations focused on choroidal vascularity in LHON even though studies have confirmed that choroidal vessels play an important role in the pathogenesis of other optic nerve diseases such as anterior ischemic optic neuropathy (AION) and glaucoma (11–14). Recent research has shed some light on changes in choroidal thickness (CT) in the eyes of LHON patients (15, 16). Nevertheless, as reported previously, the reproducibility of CT is not ideal due to its significant variation with gender, age, and refractive error. Choroidal vascularity index (CVI) is a novel and stable parameter in evaluating the choroidal vascular system and is defined as the ratio of the luminal area (LA) of the choroid to the total choroidal area (TCA) in binarized OCT images (17). In previous studies, CVI has been applied in normal subjects and patients with various ocular pathologies including AION and glaucoma (11–14). So far, CVI has not been studied in LHON patients along the natural disease course or before and after gene therapy.

The aim of the present study was to determine peripapillary CVI in the eyes of LHON patients at different stages of the disease and to explore the possible changes of CVI before and after gene therapy. Our findings help to improve our understanding of whether the choroidal vasculature is involved in the mechanisms underlying LHON pathology or gene therapy.

## Methods

### Study Subjects

This prospective multicenter clinical study (NCT03153293) was approved by the ethics committees at Tongji Hospital of Wuhan (TJ-IRB20180316), Taihe Hospital of Shiyan (2017-01), and Ezhou Central Hospital (2017-K-05) in strict accordance with the regulations of the Declaration of Helsinki. Each subject provided written informed consent prior to the examination. Participants received unilateral intravitreal injection of 0.05 mL rAAV2-ND4 (1.0 × 1,010 vg/μL) and were followed up for 6 months with complete ophthalmic and general system evaluation.

LHON patients between ages of 6 and 65 years old with confirmed genetic diagnosis of mtDNA 11,778 (G > A) mutation and binocular best-corrected VA (BCVA) worse than 0.3 logMAR were recruited at Tongji Hospital (Tongji Medical College of Huazhong University of Science and Technology). Exclusion criteria: Systemic and other ocular diseases that may affect the patient's cisual function, use of any drug or therapy that may interfere the gene therapy within 6 months (such as idebenone), heavy smokers or drinkers and pregnant women. For each subject, the eye with poorer BCVA was selected for intravitreal rAAV2-ND4 injection, while the right eye was selected if the BCVA was the same bilaterally. The BCVA improvement ≥ 0.3 logMAR was considered clinically significant.

Patients with LHON were divided into three groups based on the international consensus statement (18) and clinical practice: group 1 (active stage), disease duration ≤ 12 months; group 2 (early chronic stage), disease duration 12–24 months; group 3 (late chronic stage), disease duration ≥ 24 months. Fifteen age-matched and sex-matched healthy individuals were also recruited as healthy controls and underwent OCT examination at baseline.

### Ophthalmologic Examinations

LHON patients underwent full ophthalmologic examinations including intraocular pressure (IOP), BCVA testing, visual field, visual evoked potentials (VEP), electroretinography (ERG), and OCT examination before treatment and 1, 3, and 6 months after intravitreal injection.

### OCT Analysis

All subjects were imaged using a 3.4-mm-diameter 360-degree-circle scan centered on the optic nerve head on OCT equipment (Spectralis, Heidelberg Engineering GmbH, Heidelberg, Germany) by experienced operators. CT in each quadrant was manually determined by measuring the vertical distance between the Bruch membrane interface and the choroid-sclera interface using the built-in software.

**Figure 1:**
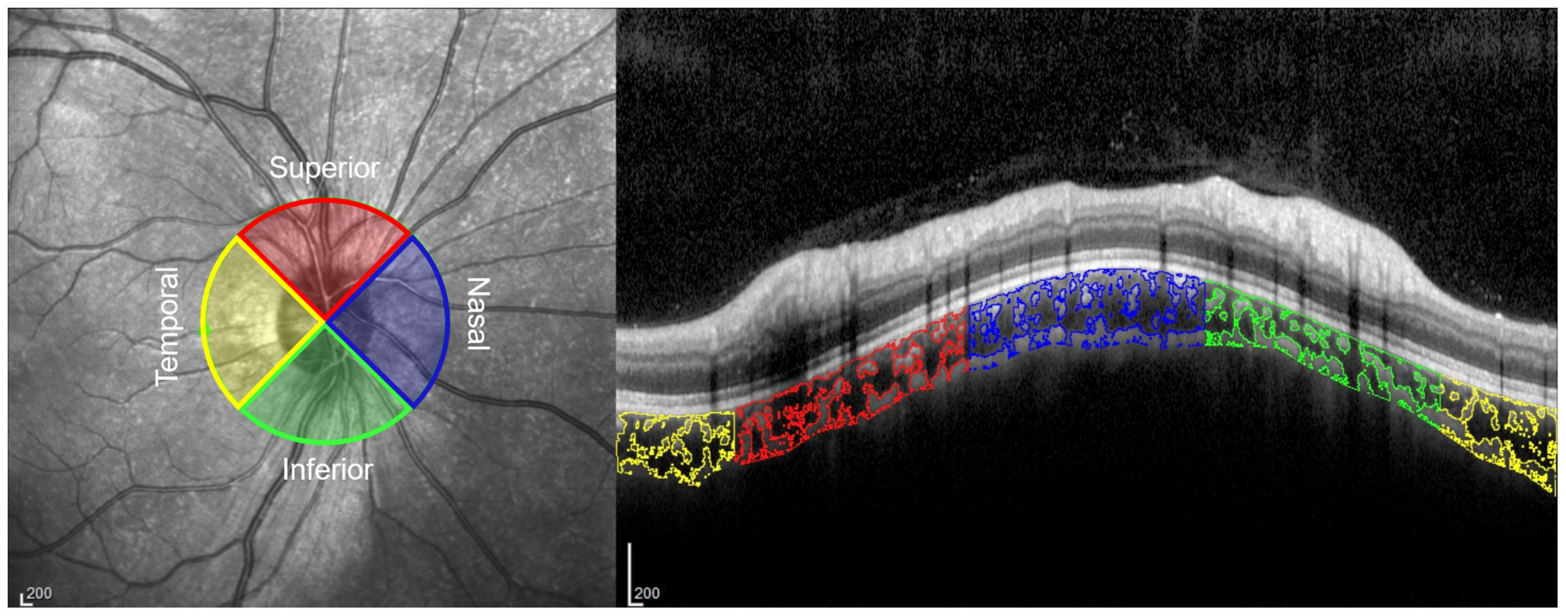
Peripapillary choroidal vascularity index (CVI) in 4 quadrants on an optical coherence tomography (OCT) image. The choroidal luminal areas in the temporal, superior, nasal, and inferior quadrants are outlined in yellow, red, blue, and green, respectively.

To measure the CVI, the OCT images were binarized and segmented using ImageJ (version 1.53c, http://fiji.sc/) as described previously (19). First, the image was opened in ImageJ and converted to an 8-bit image. Then, Niblack's auto local threshold adjustment was applied to binarize the image and the LA was determined using the threshold tool, with light pixels representing stromal area and dark pixels representing LA. Manual segmentation of TCA was done using the polygon tool after applying the pixel to mm conversion. The upper boundary of the TCA was along the Bruch membrane interface and the lower boundary was along the choroidal-scleral interface. Finally, the image was equally divided into four quadrants (superior, inferior, temporal, and nasal), and TCA, LA, stromal area, and CVI in each quadrant were then automatically calculated and recorded (Figure 1). The analysis of OCT images was conducted by two independent investigators, and the images that were judged to be of poor quality by both investigators were excluded.

### Statistical Analysis

Statistical analysis was performed using SPSS 23.0 software (SPSS Inc, Chicago, Illinois, USA). A Shapiro–Wilk test was used to evaluate the normal distribution of the variables. CT, TCA, and CVI in different duration groups at baseline were compared using one-way ANOVA. CT, TCA, and CVI at baseline and 1, 3, and 6 months after gene therapy were compared using one-way ANOVA for repeated measurements. A Bonferroni *post hoc* test was performed for pairwise comparisons. *P* < 0.05 was considered statistically significant.

## Results

### Study Population

159 LHON patients (159 Eyes) with the G11778A mutation were recruited for this study. 85 LHON patients were excluded on the basis of incomplete follow-up after 6 Months and 17 LHON patients were excluded on the basis of vague scleral junction in low-quality OCT images. Overall, 57 LHON patients were included in this study. among the 57 LHON patients, 47 (82.46%) were male, and the average age Was 16.97 ± 6.52 Years (Range: 8–41 Years). The mean BCVA was 1.66 ± 0.44 (Range: 0.7–2.3 LogMAR) and the mean IOP Was 18.93 ± 3.9 MmHg (Range: 12–28 MmHg). The mean disease duration was 34.93 ± 52.12 Months (Range: 1–312 Months). The demographic data of difference of LHON patients at different stages are shown in Table 1.

**Table 1:** Demographic and clinical characteristics of LHON Patients in different disease courses.

**Characteristic**	**Group 1** **(***N*** = 16)**	**Group 2** **(***N*** = 24)**	**Group 3** **(***N*** = 17)**
Sex (m:f)	12:4	22:2	13:4
Age (years)	14.81 ± 5.11	17.17 ± 3.56	22.17 ± 8.80
BCVA (LogMar)	1.60 ± 0.42	1.68 ± 0.46	1.68 ± 0.44
Disease duration (months)	6.38 ± 1.12	17.42 ± 3.35	86.53 ± 73.34

*LHON, leber hereditary optic neuropathy; BCVA, best-corrected visual acuity*.

### Peripapillary CT, TCA, and CVI Within Each Group at Baseline

**Figure 2:**
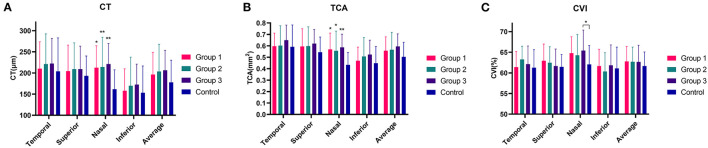
The peripapillary choroidal thickness (CT), total choroidal area (TCA) and CVI in Leber's hereditary optic neuropathy (LHON) patients in three duration groups and in normal controls **(A–C)**. Note that the CT, TCA and CVI in LHON patients were slightly higher than those in controls, especially in nasal quadrant **(A–C)**. *P < 0.05 and **P < 0.01 indicate statistically significant difference.

Table 2, Figure 2 show the peripapillary CT, TCA, and CVI of each quadrant based on OCT imaging of the eyes of patients with LHON and healthy controls. No significant difference was found in average CT between LHON patients at different stages and healthy controls (*P* = 0.468). For each quadrant, values of nasal CT in group 1–3 were significantly higher than that of healthy controls (P = 0.015, 0.007, and 0.004, respectively), while no significant difference was found in the superior, nasal, and inferior quadrants (*P* = 0.835, 0.835, and 0.747, respectively)

**Table 2:** Peripapillary CT, TCA and CVI in LHON patients of different duration groups and healthy controls at baseline.

	**Group 1**	**Group 2**	**Group 3**	**Control**	***P*** **-value**
	**(*N* = 16)**	**(*N* = 24)**	**(*N* = 17)**	**(*N* = 15)**	
**CT (μm)**					
Temporal	210.27 ± 63.47	221.17 ± 71.29	222.22 ± 59.36	203.72 ± 79.30	0.835
Superior	204.41 ± 61.67	209.33 ± 61.79	209.16 ± 54.40	193.27 ± 46.90	0.835
Nasal	212.66 ± 51.75	214.08 ± 70.44	221.03 ± 48.56	161.58 ± 45.92	0.017*
Inferior	157.96 ± 50.80	169.67 ± 67.68	172.48 ± 48.46	153.19 ± 63.45	0.409
Average **TCA (mm^2^)**	196.30 ± 52.53	203.56 ± 64.24	206.22 ± 47.23	177.94 ± 52.22	0.468
Temporal	0.60 ± 0.11	0.60 ± 0.17	0.65 ± 0.13	0.59 ± 0.19	0.697
Superior	0.59 ± 0.15	0.60 ± 0.17	0.62 ± 0.12	0.54 ± 0.13	0.547
Nasal	0.57 ± 0.14	0.56 ± 0.17	0.59 ± 0.12	0.43 ± 0.11	0.014*
Inferior	0.47 ± 0.12	0.51 ± 0.17	0.52 ± 0.13	0.45 ± 0.14	0.425
Average **CVI (%)**	0.56 ± 0.12	0.57 ± 0.15	0.59 ± 0.11	0.50 ± 0.13	0.282
Temporal	61.40 ± 3.81	63.27 ± 3.19	62.11 ± 4.41	61.26 ± 4.35	0.346
Superior	62.93 ± 4.06	62.48 ± 3.86	61.66 ± 4.13	61.48 ± 2.98	0.660
Nasal	64.77 ± 3.98	64.27 ± 5.02	65.42 ± 4.96	62.06 ± 4.59	0.224
Inferior	61.67 ± 4.07	60.35 ± 4.55	61.84 ± 4.94	61.08 ± 5.18	0.736
Average	62.80 ± 3.61	62.68 ± 3.56	62.65 ± 4.05	61.67 ± 3.39	0.812

*LHON, Leber hereditary optic neuropathy; CT, choroidal thickness; TCA, total choroidal area; CVI, choroidal vascularity index. All data are presented as mean ± SD. One-way ANOVA analysis*,

* *P < 0.05 indicates statistically significance*.

As for TCA, no significant difference was found in the values of average TCA between LHON patients at different stages and healthy controls (*P* = 0.282). For each quadrant, no significant difference was found in superior, temporal and inferior quadrants between LHON patients at different stages and healthy controls (*P* = 0.547, 0.697 and 0.425, respectively), whereas nasal TCA in groups 1–3 was significantly higher than that in healthy controls (*P* = 0.010, 0.010, and 0.003, respectively).

Although average CVI was slightly higher in LHON patients than in healthy controls, no significant difference was found in average and nasal, superior, temporal and inferior quadrants between LHON patients at different stages and healthy controls (*P* = 0.812, 0.224, 0.660, 0.346, and 0.736, respectively).

### Peripapillary CT, TCA, and CVI in LHON Patients Before and After Gene Therapy

**Figure 3:**
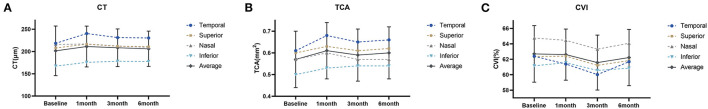
The peripapillary CT, TCA and CVI of different quadrants in LHON patients before and 1, 3, and 6 months after gene therapy **(A–C)**. Bar charts represent mean and error bars represent standard deviation. The average CT, TCA and CVI showed slight changes before and after gene therapy **(A–C)**.

Among the 57 LHON patients, 30 (52.6%) had clinically significantly improved BCVA 6 months after gene therapy compared with baseline BCVA. Table 3, Figure 3 show the peripapillary CT, TCA, and CVI of each quadrant in LHON patients before and 1, 3, and 6 months after gene therapy. The average TCA at 1 month after gene therapy was significantly higher than that before gene therapy (*P* = 0.003), while no significant difference was found in the average CT or average CVI in LHON patients before and 1,3 and 6 months after gene therapy analyzed using pairwise comparisons (all *P* > 0.05). For each quadrant, the temporal CT at baseline was significantly lower than that at 1 and 6 months after gene therapy (*P* < 0.001 and 0.033, respectively). The temporal TCA at baseline was lower than that at 1 and 6 months after gene therapy (both *P* < 0.001) and the nasal TCA at 1 month after gene therapy was higher than that at 3 and 6 months after gene therapy (*P* = 0.006 and 0.003, respectively). The superior CVI at 1 month after gene therapy was significantly higher than that at 3 months after gene therapy (*P* = 0.036). Furthermore, no significant difference was found in the maximum value of the change of average CVI between patients with improved and unimproved BCVA (*P* = 0.197).

**Table 3:** Peripapillary CT, TCA and CVI in LHON Patients Before and After Gene Therapy.

	**Baseline**	**1 m after**	**3 m after**	**6 m after**	***P*** **-value**
		**gene therapy**	**gene therapy**	**gene therapy**	
**CT (μm)**					
Nasal	215.75 ± 58.66	217.01 ± 52.65	212.42 ± 47.79	209.32 ± 45.90	0.343
Superior	207.90 ± 58.63	216.5 ± 43.57	211.78 ± 41.12	210.81 ± 40.79	0.205
Temporal	218.39 ± 64.81	240.27 ± 59.94	231.19 ± 53.23	230.38 ± 49.76	0.001*
Inferior	167.22 ± 57.50	175.71 ± 50.55	178.11 ± 46.33	177.54 ± 45.21	0.016*
Average **TCA (mm^2^)**	201.45 ± 55.67	211.17 ± 45.85	208.43 ± 42.29	206.07 ± 39.61	0.850
Nasal	0.57 ± 0.15	0.60 ± 0.14	0.57 ± 0.13	0.57 ± 0.13	0.026*
Superior	0.60 ± 0.15	0.63 ± 0.13	0.61 ± 0.13	0.62 ± 0.13	0.610
Temporal	0.61 ± 0.15	0.68 ± 0.15	0.65 ± 0.14	0.66 ± 0.15	0.001*
Inferior	0.50 ± 0.14	0.53 ± 0.14	0.54 ± 0.13	0.54 ± 0.13	0.022*
Average **CVI (%)**	0.57 ± 0.13	0.61 ± 0.13	0.59 ± 0.12	0.60 ± 0.12	0.003*
Nasal	64.76 ± 4.67	64.46 ± 4.22	63.32 ± 4.90	64.08 ± 4.69	0.118
Superior	62.36 ± 3.96	62.41 ± 3.59	61.22 ± 3.60	61.84 ± 3.41	0.024*
Temporal	62.40 ± 3.78	61.39 ± 4.84	60.02 ± 3.58	61.69 ± 3.95	0.107
Inferior	61.16 ± 4.52	61.56 ± 4.11	60.56 ± 3.57	60.85 ± 3.81	0.301
Average	62.70 ± 3.66	62.61 ± 3.31	61.58 ± 3.56	62.23 ± 3.64	0.038*

*LHON, Leber hereditary optic neuropathy; CT, choroidal thickness; TCA, total choroidal area; CVI, choroidal vascularity index. One-way ANOVA repeated measurement analysis*,

* *P < 0.05 indicates statistically significance*.

## Discussion

LHON is the most common inherited optic neuropathy and is mainly caused by mitochondrial mutations (1). Numerous mitochondria are required in the prelaminar and laminar optic nerve axons to conduct electrical signals. Consequently, the decline of ATP synthesis function caused by mitochondrial changes can result in damage to the optic nerve. The blood supply to the prelaminar and laminar optic nerve axons relies on the posterior short ciliary artery, which also provides the choroidal blood supply. It has been proposed that mitochondrial damage can affect the peripapillary vascularity through a direct effect on viability of endothelial cells and vascular smooth muscle cells (20, 21). OCTA has been used to observe the characteristics of optic nerve microvasculature in LHON. Kousal et al. (10) witnessed the dropout of peripapillary capillaries, and Balducci et al. (9) found that the microvascular changes in the temporal optic disc of LHON patients precede the changes in RNFL. Unfortunately, the choroidal vasculature was not observed in these studies.

Based on OCT imaging, previous studies have shown that CT differs in the eyes between patients at different stages of LHON progression and healthy controls (15, 16). Conversely, no significant difference in average CT was observed in our analysis. One possible reason for this discrepancy is that CT can be easily affected by various physiological factors. Current evidence indicates that compared with CT, CVI is a novel and relatively stable parameter that enables more accurate quantification of choroidal vascularity (22). In the present study, although no statistically significant difference was found in the average peripapillary TCA or CVI within the three duration groups of LHON patients and the healthy controls, the average TCA and CVI were slightly higher in LHON patients in each stage than in healthy controls. A possible reason for this result could be the relatively small sample size of patients in this study. Therefore, a follow-up with more LHON patients from across the natural course of disease progression, especially those in the subacute or dynamic stages, is essential for further research. Additionally, whether there is a difference in CVI between NA-AION patients and healthy controls still remains controversial (11, 12), indicating that CVI in optic nerve diseases has not been fully understood and more investigations are essential.

In LHON patients, the 11778G > A mutation impacts a crucial amino acid in the NADH dioxygenase subunit 4 complex 1 (ND4) gene, and therefore, intravitreal injection of rAAV2/2-ND4 is an effective method for the treatment of LHON (3). Both our previous and the current studies have shown the remarkable effectiveness of gene therapy (2, 3), while the anatomical effect of gene therapy on LHON has not been fully described yet. John et al. (23) witnessed that the temporal RNFL was thicker in treated than in fellow eyes at month 12, but due to the small sample size, more evidence is needed to further confirm whether gene therapy can reduce RNFL loss. Studies focused on choroid in LHON patients have shown that choroidal vascularity may be used as a follow-up indicator of the effectiveness of treatment (15, 16). Recent studies have demonstrated that changes in CVI can serve as a potential indicator for evaluation of treatment for various diseases (17). In our study, both CT and TCA in nasal or inferior quadrant increased after gene therapy, while CVI did not show significant changes. The possible reason for the changes of CT and TCA could be that choroidal stromal and luminal area may have a similar increase to some extent after gene therapy, but since CVI is the more stable parameter that tells the choroidal vascularity more accurately, the result may still indicate that there is no significant change in peripapillary choroidal vascularity after gene therapy. In addition, there was also no significant difference in the maximum value of the change in average CVI between patients with improved and unimproved BCVA. Our findings raise the possibility that choroidal vascularity may not be highly involved in the mechanisms underlying the gene therapy of LHON, but larger scale research is still required to confirm this hypothesis in the future.

The present study has some limitations. The sample size was limited due to the low incidence of the disease, and the follow-up time for patients after gene therapy was relatively short. Studies with larger sample sizes and longer follow-up time are still essential. Considering the loss of follow-up and the low-quality images caused by poor VA in some patients, there is still population bias in our study. The relationship between macular CT and anatomical changes in LHON patients at different stages of disease progression has been described in previous research. The observation on macular CVI is therefore also of great value and will be carried out in future work. Furthermore, the investigation of the relationship between peripapillary CVI, retinal capillaries, and choroidal capillaries (CC) observed by OCTA, as well as the exploration of the possible correlation between CVI and the visual prognosis of LHON patients are promising avenues for future study.

Overall, to our knowledge, our study is the first to investigate CVI in LHON patients. No significant difference was found in peripapillary choroidal vascularity within LHON patients at different stages and healthy controls, and choroidal vascularity seems to stay stable after gene therapy. Still, future studies at a larger scale are still necessary.

## Data Availability Statement

The raw data supporting the conclusions of this article will be made available by the authors, without undue reservation.

## Ethics Statement

The studies involving human participants were reviewed and approved by the Ethics Committee at Tongji Hospital of Wuhan, Taihe Hospital of Shiyan, and Ezhou Central Hospital. Written informed consent to participate in this study was provided by the participant's legal guardian/next of kin.

## Author Contributions

JY and CC: conceptualization and study design. JJ and GS: data collection, methodology, and writing. QM and DW: data collection and analysis. BL and JY: data provisiong. CC: review and editing. All authors contributed to the article and approved the submitted version.

## Funding

This article was supported by the National Natural Science Foundation of China (Grant No. 82101115).

## Conflict of Interest

BL was employed by the company Neurophth Biotechnology. The remaining authors declare that the research was conducted in the absence of any commercial or financial relationships that could be construed as a potential conflict of interest.

## Publisher's Note

All claims expressed in this article are solely those of the authors and do not necessarily represent those of their affiliated organizations, or those of the publisher, the editors and the reviewers. Any product that may be evaluated in this article, or claim that may be made by its manufacturer, is not guaranteed or endorsed by the publisher.
